# Immunohistochemical expression of CD117/KIT, HER2, and Erβ in schistosomal and non-schistosomal urothelial carcinoma of Egyptian patients

**DOI:** 10.1007/s11255-023-03667-1

**Published:** 2023-06-20

**Authors:** Sara E. Khalifa

**Affiliations:** Department of Pathology, Faculty of Medicine, Cairo University, Cairo, 11728 Egypt

**Keywords:** Schistosomiasis, CD117/KIT, HER2, ERβ

## Abstract

Bladder carcinoma is an endemic problem in Egypt with schistosomiasis being an additional risk factor. Due to gender disparities, Erβ investigation and its role in modulating chemosensitivity are studied. CD117/KIT expression is also considered since the emergence of the targets of the tyrosine kinase inhibitor imatinib mesylate (Gleevec). HER2 is one of the established therapeutic targets in many cancers. We aimed to investigate CD117/KIT immunoexpression in schistosomal and non-schistosomal urothelial carcinoma of Egyptian patients and its relationship with HER2 and Erβ expressions, correlating it with pertinent variables that will help to provide better treatment options of possible combined targeted and hormonal therapy that might be effective against this aggressive malignancy. Sixty cases of bladder carcinoma were tested. Depending on the schistosomiasis association status of each case, two groups have been established with 30 cases each. CD117/KIT, HER2, and ERβ immunostaining were done and correlated with clinico- immuno-pathological parameters. CD117/KIT expression was seen in 71.7% of cases that correlated significantly with schistosomiasis (*P* = 0.01). In addition, a positive correlation was detected between schistosomiasis association and the percentage of immunostained cells and intensity score of CD117/KIT with *P* = 0.027, 0.01, respectively. 30% and 61.7% of cases were positively stained with HER2 and Erβ, respectively, with no significant relation with schistosomiasis. Due to the high expression, we found further clinical trials are needed to offer individualized targeted therapeutic options in urothelial tumors using anti-CD117/KIT, HER2, and ERβ other than limited traditional chemo- and nontargeted therapies.

## Introduction

Bladder cancer is a significant problem in Egypt, where Egypt ranks 10th in the worldwide incidence of bladder cancer for both sexes [1], accounting for 9.4% of all cases of cancer. Male predominance was noted as the male-to-female ratio is 3:1. In males, bladder carcinoma is considered 2nd most common malignancy after liver cancer [2]. An additional risk factor in the Middle East and parts of Africa is schistosomiasis. In Egypt, it is an endemic problem dating back to the times of the Pharaohs, since calcified eggs were found in some mummies [3]. In recent years, there is a change in the spectrum from squamous carcinoma to urothelial carcinoma with only limited data on this specific type of cancer.

Gender disparities have prompted an investigation of sex hormones and their receptors in bladder cancer. ER (which is now called ERα) and ERβ are physiologically expressed in a variety of human organs and have a variety of functions in these tissues when estrogens such as 17-estradiol (E2) bind to them. [4]. In preclinical models for several types of endocrine malignancies, such as breast, ovary, and prostate carcinomas; ERα and ERβ have also been shown to function differently. In addition, there is an increasing amount of evidence to suggest the involvement of estrogen-mediated ER signaling in the development and progression of urothelial cancer. ER activation has also been associated with one of the molecular subtypes, the luminal subtype, in muscle-invasive bladder cancer [5]. Estrogen receptors, including ERα and ERβ, have been shown to contribute to urothelial carcinogenesis and cancer progression, as well as modulating chemosensitivity in bladder cancer [4].

Despite the anticipated radical treatment of the primary lesion of urinary bladder carcinoma, approximately half of the patients with the muscle-invasive disease die within 5 years of diagnosis. These cases are assumed to have micrometastasis already preoperatively and cannot be identified during initial staging [6]. Thus, there is a need for improved methods of diagnosis and therapy in muscle-invasive cases.

Targeted tumor therapy is based on the delivery of cytotoxic substances directly to the tumor cell, with minimal damage and influence on surrounding normal tissue. The optimal target is located on the cell surface and expressed exclusively on tumor cells. However, this is rarely the case, and therefore ‘normal’ target structures, expressed at a significantly higher level on tumor cells than normal cells, have to be used [7].

The proto-oncogene c*-kit* encodes a transmembrane tyrosine kinase growth factor receptor protein (CD117/KIT) that is structurally related to the platelet-derived growth factor/colony-stimulating factor-1 (PDGF/CSF-1) subfamily [8]. In terms of therapeutic strategies, it has become important to evaluate the frequency of CD117/KIT expression and mutation in malignancies since the emergence of the targets of the tyrosine kinase inhibitor imatinib mesylate (Gleevec) [9] a potential therapeutic agent which acts by specifically inhibiting tyrosine kinase receptor that is characteristic of a particular cancer cell, rather than non-specifically inhibiting and killing all rapidly dividing cells [10].

HER2 has been viewed as a protein of potential prognostic importance in addition to being a therapeutic target with involvement in the pathogenesis of numerous human cancers [11]. Expression of HER2 has been recognized in some tumor types mainly breast, gastric, oesophageal, pancreatic, and many others. Even though there is agreement about antibodies and tyrosine kinase inhibitors targeting HER2 in the breast, gastric and esophageal adenocarcinomas, still there are other evident prospects in other tumor types [12].

In the present study, we investigated the expression of CD117/KIT in schistosomiasis-related urinary bladder carcinoma from Egyptian patients and non-schistosomiasis related urinary bladder carcinomas and its relationship with immunohistochemical expression of HER2 and ERβ and attempted to correlate it with pertinent variables that will help to provide better treatment options of possible combined targeted and hormonal therapy that might be effective against this aggressive malignancy.

## Materials and methods

This retrospective study was conducted on 60 cases of bladder carcinoma. All procedures performed were in accordance with the ethical standards of the Kasr Alainy Research Ethics Committee (REC) that operates according to ICH GCP guidelines and applicable local and institutional regulations and guidelines which govern REC operation with a ref no. (N-1192023).

Formalin-fixed paraffin-embedded tumor (FFPE) blocks were collected from the Pathology Department, Kasr Alainy Hospital, Faculty of Medicine, Cairo University. They included 46 cases of radical cystectomy and 14 cases of TUR (all cases of TUR are non-muscle invasive).

Demographic and clinical data of all cases were summarized from the patients’ files. Exclusion criteria for tumor blocks included scanty tumor tissue, poor fixation, overt necrosis, and Cases with missing data. Findings were then tabulated.

The clinicopathological characteristics of our patients are summarized in Table 1.

**Table 1:** Schistosomiasis association in correlation to urinary bladder carcinoma patients’ clinicopathologic characteristics and immunohistochemical expressions

	Schistosomiasis-related urinary bladder carcinoma group (+) 30 cases		Control group (−) 30 cases		Total		P
CD117/KIT							
Staining expression							
Positive	26 (86.67%)		17 (56.67%)		43 (71.7%)		0.010
Negative	4 (13.33%)		13 (43.33%)		17 (28.3%)		
Percentage of stained cells							
> 50%	19 (63.33%)		11 (36.67%)		30 (50%)		0.027
10–50%	7 (23.33%)		6 (20%)		13 (21.67%)		
< 10%	4 (13.33%)		13 (43.33%)		17 (28.3%)		
Intensity score							
3	6 (20%)		0 (0.0%)		6 (10%)		0.001
2	20 (66.7%)		4 (13.33%)		24 (40%)		
1	0 (0.0%)		13 (43.33%)		13 (21.7%)		
0 (−)	4 (13.33%)		13 (43.33%)		17 (28.3%)		
HER2							
Positive (score 3+)	10 (33.33%)		8 (26.67%)		18 (30%)		0.735
Equivocal (score 2+)	7 (23.33%)		8 (26.67%)		15 (25%)		
Negative							
Score 1+	13 (43.33%)	8 (26.67%)	14 (46.67%)	6 (20%)	27 (45%)	14 (23.33%)	
Score 0		5 (16.67%)		8 (26.67%)		13 (21.67%)	
ER ß							
Positive	19 (63.33%)		18 (60%)		37 (61.67%)		0.500
Negative	11 (36.67%)		12 (40%)		23 (38.33%)		
Sex							
Male	27 (90%)		26 (86.67%)		53 (88.33%)		0.500
Female	3 (10%)		4 (13.33%)		7 (11.67%)		
Morphologic variability							
Non-invasive							0.051
Papillary	1 (3.33%)		8 (26.67%)		9 (15%)		
CIS	1 (3.33%)		1 (3.33%)		2 (3.33%)		
Invasive							
Tcc, conventional	18 (60%)		12 (40%)		30 (50%)		
Tcc with squamoid differentiation	9 (30%)		5 (16.67%)		14 (23.33%)		
Tcc with glandular differentiation	1 (3.33%)		3 (10%)		4 (6.67%)		
Tcc with micropapillary differentiation	0 (0.0%)		1 (3.33%)		1 (1.67%)		
Grade							
High	26 (86.67%)		23 (76.67%)		49 (81.67%)		0.253
Low	4 (13.33%)		7 (23.33%)		11 (18.33%)		
T stage							
Ta	1 (3.33%)		8 (26.67%)		9 (15%)		0.054
Tis	1 (3.33%)		1 (3.33%)		2 (3.33%)		
T1	2 (6.67%)		4 (13.33%)		6 (10%)		
T2	11 (36.67%)		4 (13.33%)		15 (25%)		
T3	13 (43.33%)		10 (33.33%)		23 (38.33%)		
T4	2 (6.67%)		3 (10%)		5 (8.33%)		

Depending on the schistosomiasis association status of each case, two groups have been established with 30 cases each.

4 μm sections from each submitted tumor paraffin block were stained with hematoxylin and eosin, to assess pertinent histologic findings.

### Immunohistochemical staining

Serial sections (4 μm) were additionally prepared and float-mounted on adhesive-coated glass slides for immunohistochemical staining.

Anti-CD117(ckit) antibody (A4502, DAKO, Dako Corporation, Carpinteria, CA, USA) at 1:200 dilution was used for CD117/KIT and primary antibodies included rabbit antihuman c-erbB2 (HER2) oncoprotein antibody (DAKO, Dako Corporation, Carpinteria, CA, USA) at 1:200 dilution for HER2. In addition, rabbit antihuman estrogen receptor beta antibody, diluted at 1:100 (GTX23577, Gene Tex, USA) were used for ERβ. The DAKO Envision system (DAKO Envision labeled polymer, peroxidase) was used as the detection system.

Control slides of selected cases of GIST were run in parallel, in case of staining with anti-CD117(ckit) antibody, and the positive control for HER2 was a known HER2-overexpressing breast carcinoma. Sections of prostatic adenocarcinoma were used as a positive control in case of ERβ antibody. A negative control was obtained for all cases by omitting the primary antibodies.

### Evaluation of the staining

Distinct membranous and cytoplasmic CD117/KIT immunostaining expression was evaluated as positive. The cutoff value of 10% was accepted as positive staining. Percentage of stained cells was evaluated, scored as (1) when 10–50% of the cells were stained, and (2) when > 50% of the cells were stained. Staining intensity was evaluated only in positive cases and scored as (1) for weak staining (faint, light yellow), (2) for moderate staining (brown), and (3) for strong staining (dark brown) [13].

Only nuclear staining for ERβ was considered to be positive. German immunoreactive score was calculated by multiplying the percentage of immunoreactive cells (0% = 0; 1–10% = 1; 11–50% = 2; 51–80% = 3; 81–100% = 4) by staining intensity (0, negative; 1, weak; 2, moderate; 3, strong). Scores ranges from 0 to 12. Any score between 0 and 2 was considered as negative and any score above 2 was considered to be positive [14].

To determine the score of HER2 expression, both the membrane and cytoplasmic staining patterns were estimated, and its intensity was scored on a scale of 0 to 3+ .

Scoring of the immunohistochemical staining for HER2 overexpression was performed according to what was published by the ASCO/CAP. According to this joint document, a positive HER2 result is: immunohistochemical staining of 3+ (uniform, intense circumferential membrane staining of greater than 10% of invasive tumor cells). A negative HER2 result is: immunohistochemical staining of 0 (No staining or faint, incomplete membrane staining in ≤ 10% of tumor cells) or 1+ (incomplete membrane staining that is faint/barely perceptible and within > 10% of tumor cells), an equivocal HER2 result is: immunohistochemical staining of 2+ (weak and moderate complete membrane staining in greater than 10% of tumor cells) [15, 16].

### Statistical analysis

SPSS (statistical product for services solutions, version 22.0, IBM Corporation, New York, USA) was used and correlations were determined using the *χ*^2^-test. A *P* value of less than 0.05 was chosen to represent statistical significance.

## Results

Sixty bladder carcinoma cases were enrolled in this study. Median age was 61 years, with 53 (88.3%) males and 7 (11.7%) females. Patients ranged in age from 25 to 81 years, with a mean of 62 ± 10.766 years.

All the 60 cases included in both groups were evaluated for CD117/KIT, HER2, and ER ß expression by immunohistochemical staining.

Distinct membranous and cytoplasmic CD117/KIT immunostaining expression was seen in 71.7% of cases (43 out of 60 cases). Thirty out of the positive 43 showed expression in more than 50% of the neoplastic cells while 13/43 expressed staining just in 10–50% of the cells. Strong staining (dark brown color, score 3) was observed in 6 out of the 43 positive cases (13.95%) while 24 cases out of 43 positive cases (55.81%) showed moderate staining (brown color, score 2); and 13 cases (30.23%) displayed weak staining (faint or light blue color, score 1). On the other hand, no staining or staining of less than 10% of tumor cells was detected in 17 out of the whole 60 cases (28.3%).

**Fig. 1:**
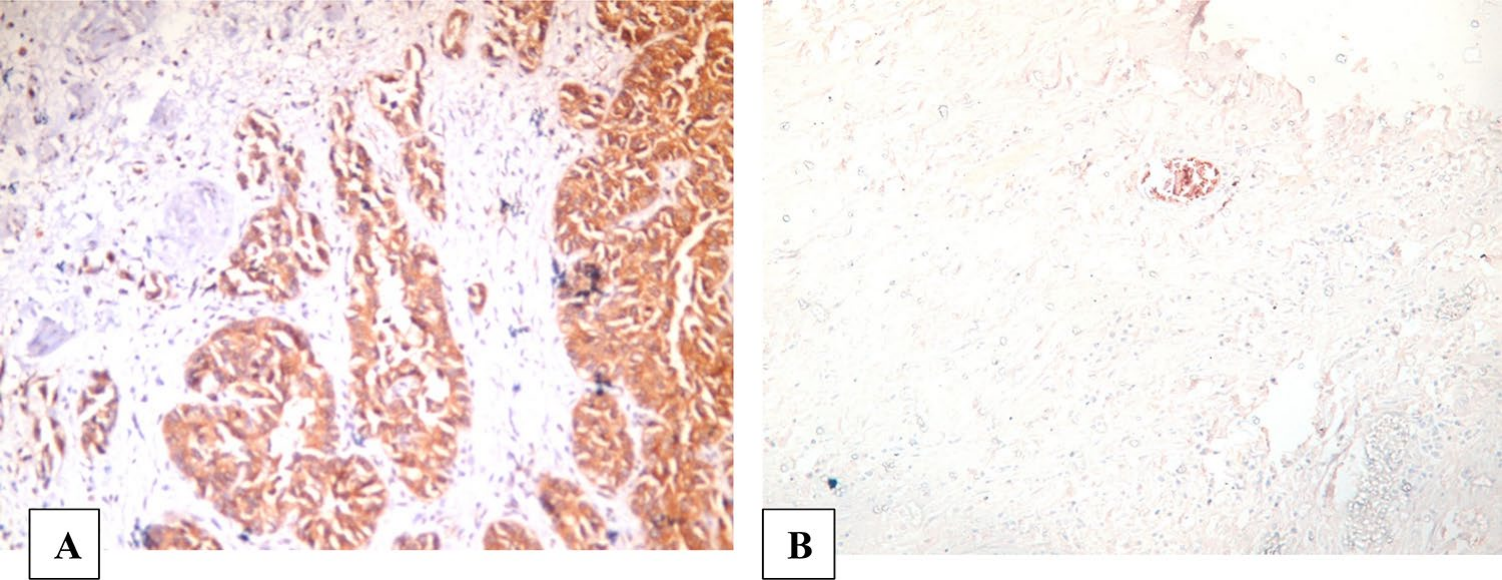
**A** High-grade urothelial carcinoma, showing strong CD117/KIT cytoplasmic expression in more than 50% of tumor cells (original magnification, CD117/KIT, × 400). **B** Negative expression of CD117/KIT in urothelial carcinoma associated with schistosomiasis (original magnification, CD117/KIT, × 400)

Immunohistochemical CD117/KIT overexpression correlated significantly with schistosomiasis association with a *P* value = 0.01, where 26 cases (86.67%) out of the 30 schistosomiasis-related urinary bladder carcinoma and 17 (56.67%) out of 30 control (non-schistosomiasis related urinary bladder carcinomas were CD117/KIT positive. A significant positive correlation was detected between schistosomiasis association and CD117/KIT percentage of immunohistochemically stained cells (*P* = 0.027), as more than half of cases with schistosomiasis association (19/30 cases, 63.3%) showed widely extensive positive staining in more than 50% of the neoplastic cells. In addition, significant variation was found relating CD117/KIT intensity score to tumoral schistosomiasis association with a *P* value of 0.001, as all the six cases with strong staining (dark brown color, score 3) were included in the schistosomiasis-related urinary bladder carcinoma group and all the 13 cases that were weakly stained (faint or light blue color, score 1) were included in the control group (Fig. 1).

Regarding HER2 immunohistochemical expression, 30% of specimens (18/60) exhibited intense circumferential membrane staining of greater than 10% of invasive tumor cells (score 3+ , positive). Weak and moderate complete membrane staining in > 10% of tumor cells (score 2+ , equivocal) was observed in 15 out of 60 cases (25%), while the vast majority of specimens exhibited negative staining (27 cases out of 60, 45%), (score 0: 23/60, 21.7% and score 1+ : 14/60, 23.3%).

**Fig. 2:**
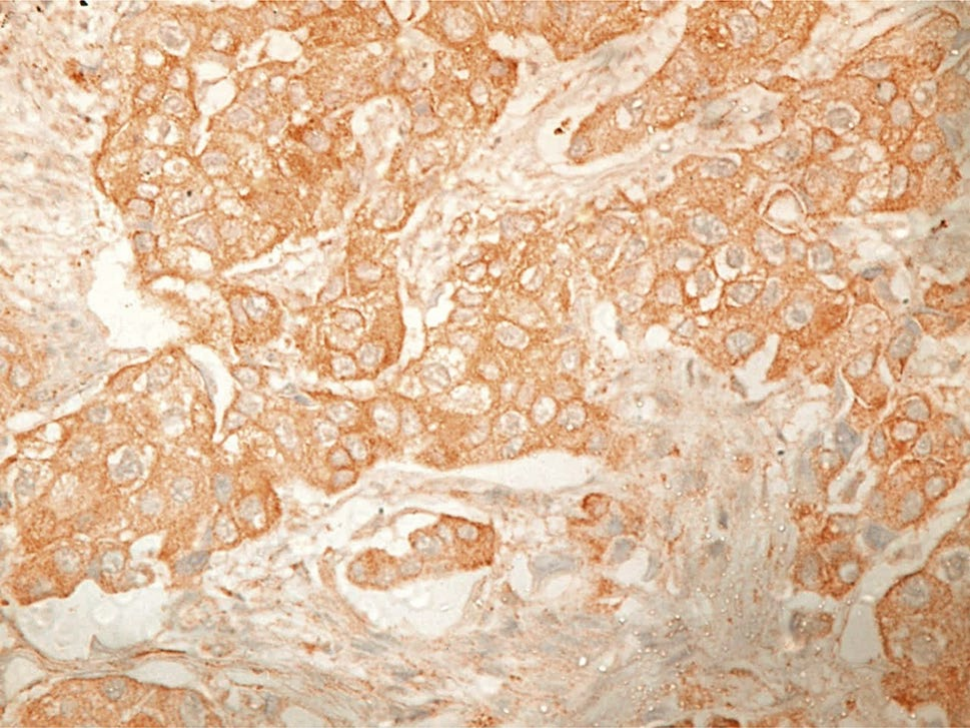
High-grade invasive urothelial carcinoma showing cytoplasmic and strong complete membrane staining for HER2 in more than 10% of tumor cells (original magnification, HER2, × 400)

33.33% (10/30) of the 30 schistosomiasis-related urinary bladder carcinomas and 8 cases (26.67%) out of 30 of the control group were HER2 positive (Fig. 2).

Of all the tumor specimens, 37 (61.7%) cases showed tumor positivity, whereas 23 (38.3%) cases were negative for ERβ staining.

**Fig. 3:**
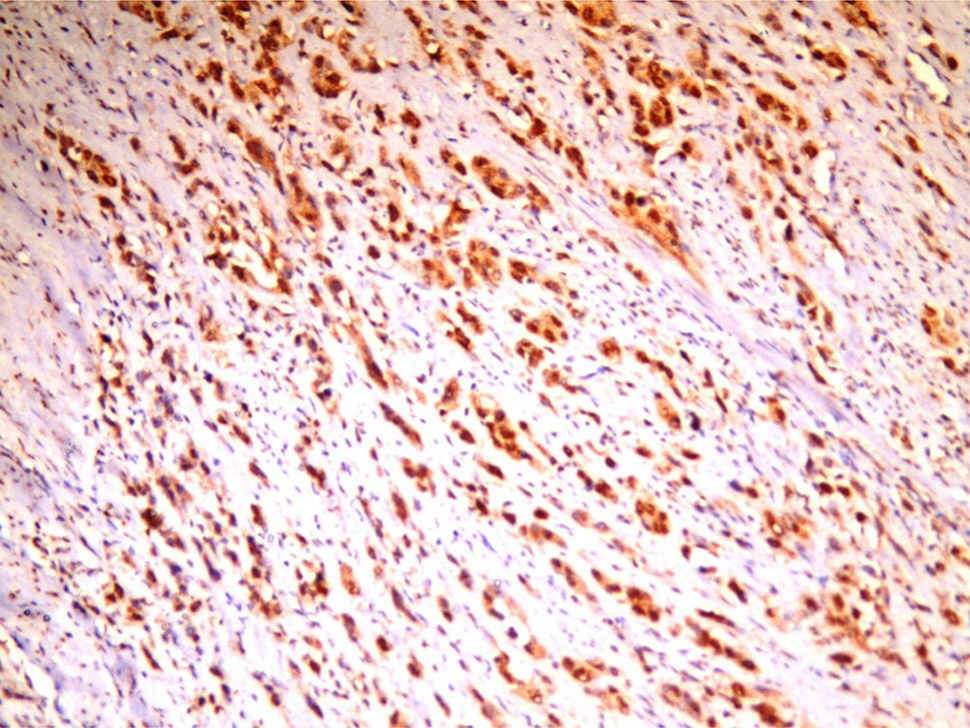
High-grade invasive urothelial carcinoma showing strong positive nuclear ERβ staining in more than 80% of the tumor cells (original magnification, ERβ, × 200)

Near equal expression of ERβ was observed in both studied groups regarding schistosomiasis association as 19/30 (63.33%) of the schistosomiasis-related urinary bladder carcinoma group and 18/30 (60%) of the control group were ER ß positive (Fig. 3).

A significant correlation was also detected between ERβ expression and sex distribution, where ERβ expression was seen in most of male patients (30 out of 53 males), *P* = 0.027.

Associations between expressions of CD117/KIT in relation to other immunohistochemical markers of urinary bladder carcinoma cases are displayed in Table 2.

**Table 2:** CD117/KIT expression in relation to other marker staining of urinary bladder carcinoma cases

		CD117/KIT staining expression		Total	*P* value
		Positive (43 cases)	Negative (17 cases)		
HER2	Positive	15 (34.9%)	3 (17.6%)	18 (30%)	0.314
	Equivocal	11(25.6%)	4 (23.5%)	15 (25%)	
	Negative	17 (39.5%)	10 (58.8%)	27 (45%)	
ER ß	Positive	27 (62.8%)	10 (58.8%)	37 (61.67%)	0.500
	Negative	16 (37.2%)	7 (41.2%)	23 (38.33%)	

No significant correlation was detected between ERβ immunohistochemical expression in bladder carcinoma cases and HER2 expression where *P* = 0.467.

CD117/KIT, HER2, and ERβ immunohistochemical expression profiles in relation to urothelial carcinoma patients’ clinicopathologic characteristics are displayed in Table 3

**Table 3:** CD117/KIT, HER2, and ERß immunohistochemical expression in relation to urothelial carcinoma patients’ clinicopathologic characteristics

		CD117/KIT		*P*	HER2		*P*	ERß		*P*
		Positive 43 (71.7%)	Negative 17 (28.3%)		Positive or equivocal 33 (55%)	Negative 27 (45%)		Positive 37 (61.67%)	Negative 23 (38.33%)	
Sex	Male	38 (63.3%)	15 (25.0%)	0.648	29 (48.3%)	24 (40%)	0.614	30 (50%)	23 (38.3%)	**0.027**
	Female	5 (8.3%)	2 (3.3%)		4 (6.7%)	3 (5%)		7 (11.7%)	0 (0%)	
Morphologic variability	Papillary	4 (6.7%)	5 (8.3%)	0.154	5 (8.3%)	4 (6.7%)	0.400	6(10%)	3(5%)	0.806
	CIS	1 (1.7%)	1 (1.7%)		0 (0.0%)	2 (3.3%)		1 (1.7%)	1 (1.7%)	
	Conventional	21(35%)	9 (15%)		16 (26.7%)	14 (23.3%)		17 (28.3%)	13 (21.7%)	
	With squamoid differentiation	13 (21.7%)	1 (1.7%)		8 (13.3%)	6 (10%)		10 (16.7%)	4 (6.7%)	
	With glandular differentiation	3 (5%)	1 (1.7%)		3 (5%)	1 (1.7%)		2 (3.3%)	2 (3.3%)	
	With micropapillary differentiation	1 (1.7%)	0 (0.0%)		1 (1.7%)	0 (0.0%)		1 (1.7%)	0 (0.0%)	
Grade	High	36 (60%)	13 (21.7%)	0.377	26 (43.3%)	23 (38.3%)	0.385	30 (50%)	19 (31.7%)	0.583
	Low	7 (11.7%)	4 (6.7%)		7 (11.7%)	4 (6.7%)		7 (11.7%)	4 (6.7%)	
T stage	Ta	4 (6.7%)	5 (8.3%)	0.164	5 (8.3%)	4 (6.7%)	0.222	6 (10%)	3 (5%)	0.100
	Tis	1 (1.7%)	1 (1.7%)		0 (0.0%)	2 (3.3%)		1 (1.7%)	1 (1.7%)	
	T1	3 (5%)	3 (5%)		4 (6.7%)	2 (3.3%)		1 (1.7%)	5 (8.3%)	
	T2	11 (18.3%)	4 (6.7%)		10 (16.7%)	5 (8.3%)		9 (15%)	6 (10%)	
	T3	20 (33.3%)	3 (5%)		10 (16.7%)	13 (21.7%)		18 (30%)	5 (8.3%)	
	T4	4 (6.7%)	1 (1.7%)		4 (6.7%)	1 (1.7%)		2 (3.3%)	3 (5%)	

Bolded value indicates statistical significance

## Discussion

Despite the leading etiology of bladder carcinomas in Western countries being mainly due to smoking and occupational exposures, chronic bladder infection with schistosomiasis has been documented as the most important risk factor for bladder cancer in Egypt [17]. Searching for prognostic markers in both Schistosomal and non-Schistosomal urinary bladder carcinomas is essential for possible targeting and therapeutic intervention. In the current study, we investigated the most common targetable biomarkers in a series of urinary bladder carcinoma to identify treatment options that are not typically considered in this population. We first examined CD117/KIT expression in schistosomal and non-schistosomal urinary bladder carcinomas from 60 Egyptian patients. We then demonstrated the association between its expression profiles and immunohistochemical expression of HER2 and ERβ, then addressed our finding of immunohistochemical expressions correlating them with all available clinicopathologic parameters.

We found that 43 out of our 60 cases (71.1%) showed CD117/KIT expression, patients with positive bilharzial ova expressed CD117/KIT higher than negative patients (86.67% vs. 56.67%, respectively, showing significant association, *P* = 0.01). In addition, a statistically significant positive correlation was detected between schistosomiasis association and CD117/KIT percentage of immunohistochemically stained cells (*P* = 0.027), as19/30 (63.3%) of schistosomal urinary carcinomas showed widely extensive positive staining in more than 50% of the neoplastic cells. Moreover, significant variation was found relating CD117/KIT intensity score to tumoral schistosomiasis association with a *P* value of 0.001, as all the six cases with strong staining (dark brown color, score 3) were included in the bilharzial urinary bladder carcinoma group and all the 13 cases that were weakly stained (score 1) were included in the control group. Our findings are consistent with what was stated by Shams et al. [18] and similarly supported by Went et al. [19] who assured CD117/KIT upregulation in urinary bladder SCCs. Moreover, a previous study on urinary bladder small cell carcinomas demonstrated CD117/KIT overexpression in 27% of the studied cases [20].

In bladder urothelial carcinomas, HER2 status has been intensively investigated, with varied results on the prevalence of HER2 expression (ranging, from 0 to 42%). it was estimated to be around 6% in the two largest studies, done by Lae et al. [21] and Millis et al. [22]. According to our findings, HER2 expression was found in 30% of urinary carcinoma cases. Our work’s significantly greater HER2 positivity could be due to the predominately advanced urothelial carcinomas we looked at. Similarly, Salem et al. [23] and Badary et al. [24] reported higher rates of HER2 positivity.

HER2 positivity was found in 33.33% of the schistosomiasis-related urinary bladder carcinomas cases and 26.67% of the control cases with no statistically significant association (*P* = 0.735). Our findings were in accordance with Salem et al. [23] who reported no significant correlation between concomitant bilharzial cystitis and the expression of HER2, and with what was stated by Hammam et al. [25] that bilharzial infestation seemed not to affect the HER2 expression.Of our cases, 61.7% showed ERß tumor positivity. Our findings agreed with those reported by Kontos et al. [26], who found that 55.2% of patients were ER ß positive. This was supported by Ide et al. [27], who discovered in their meta-analysis study that ERß expression in urothelial tumor tissues ranged from 22 to 100%. Another meta-analysis done by Goto and Miyamoto [4] revealed that ERß expression has been reported to be positive in 27–100% of urothelial tumors. These variations could be due to changes in antibody specificity or staining techniques and tissue preparation, such as fixative preservation.

Near equal expression of ERß was observed in both studied groups regarding schistosomiasis association as 63.33% of the schistosomiasis-related urinary bladder carcinoma group and 60% of the control group were ERβ positive with no statistically significant correlation (*P* = 0.5) This disagrees with Hakim [28] who found a statistically significant decline in the immunohistochemical expression of ERβ in urothelial carcinoma associated with schistosomiasis (*P* value 0.001).We also found a significant correlation between ERβ expression and sex distribution, with ERβ expression seen in the majority of male patients (30 out of 53 males) as *P* value = 0.027. This was consistent with the findings of the meta-analysis done by Ide et al. [27] who noted that ERß expression has been reported to be positive more in men than in women.

We did not find a statistically significant correlation either between expressions of CD117/KIT in relation to HER2 and ERβ immunohistochemical positivity or between ERβ immunohistochemical expression in bladder carcinoma cases and HER2 expression where *P* > 0.05. Our findings were following that of Millis et al. [22] who affirmed that except for the favorable correlation with EGFR, no other biomarker demonstrated a meaningful difference in HER2 status.

We found a near statistically significant correlation regarding schistosomiasis association with T staging, *P* = 0.054. This agrees with Wishahi et al. [29] who found Schistosoma antigen overexpression in muscle-invasive bilharzial cancer but was negative in the non-muscle invasive bladder cancer group and the non-schistosomal muscle-invasive group.

Since the assessment of markers in combination may perform better than those considered individually, therefore, in order to emphasize the prognostic value of new targeted therapeutic agent in bladder urothelial carcinoma, we immunohistochemically examined the expression of CD117/KIT, HER2, and ERβ in 2 groups of Egyptian patients suffering from urothelial carcinoma either schistosomally related or not. Our data from this combined analysis could help us to develop new therapeutic approaches that selectively antagonize CD117/KIT, HER2, and ERβ, or act upon the downstream target genes of these receptors that might be applied individually or in combination to target bladder cancer. However, further studies are still needed to support these findings.

## Conclusion

Without a doubt, schistosomiasis is a major health concern in Egypt. Recognizing some morphological features and subtypes of urothelial carcinoma could be a useful prognostic factor in treatment planning; thus, schistosomal/urothelial bladder cancer should be considered a histological variant being positively correlated with T staging and CD117/KIT overexpression.

Using immunohistochemistry, the present study demonstrated a high expression of CD117/KIT, HER2, and ERβ in the urinary bladder urothelial carcinoma. The relatively high percentage of their expression (71.1%, 30%, and 61.7%, respectively) indicates that there is a sizable group of bladder cancer patients who might potentially benefit from targeted therapy and underlines the need for further clinical trials to offer individualized targeted therapeutic options other than limited traditional chemo- and nontargeted therapies.

## Data Availability

The data supporting the findings of the study are available within the article. The data supporting the findings of the article is available in the Kasr Alainy pathology department files at https://medicine.cu.edu.eg/index.php/en/.
